# Study on the application of 3D printing head film fixation technology in cranial radiotherapy

**DOI:** 10.7150/jca.82909

**Published:** 2023-04-09

**Authors:** Wei Guo, Bin Wang, Li-Yuan Zhang, Yun-Chuan Sun, Tao Xue

**Affiliations:** 1 Department of Radiation Oncology, Hebei Province Cangzhou Hospital of Integrated Traditional and Western Medicine, Cangzhou Hebei, 061000, China.; 2 Key Laboratory of Endocrinology of National Health Commission, Department of Endocrinology, State Key Laboratory of Complex Severe and Rare Diseases, Peking Union Medical College Hospital, Chinese Academy of Medical Science and Peking Union Medical College, Beijing, 100730, China.

**Keywords:** 3D printing, traditional thermoplastic, head film, cranial radiotherapy, CBCT

## Abstract

**Objective:** To investigate the use of 3D printing technology to customize individualized precise radiotherapy head masks for cranial radiotherapy patients. Through the comparison with thermoplastic head film, evaluate the effect of this material on deep dose attenuation and body surface dose, and evaluate its positioning accuracy and repeatability for clinical application.

**Methods:** Thirty patients with head and neck radiotherapy were divided into the control group and the experimental group. The control group used the traditional thermoplastic head film fixation technique for body position fixation, and the experimental group used the 3D printing head film fixation technique. The patient setup was verified by kV-CBCT scanning to obtain the translational setup error and rotational setup error in the X, Y, and Z directions.

**Results:** At a depth of 5 cm, both materials have a radiation attenuation rate of <1%. At the surface location, the body surface dose of control group increased by approximately 27%. With a 3D printing head film, the body surface dose increased by approximately 18%. The positioning of two groups of patients was verified by the kV-CBCT, and a total of 232 data sets were obtained. The average translation positioning errors in the X, Y, and Z direction of control group and experimental group were 1.29 mm, 1.42 mm, 1.38 mm and 1.16 mm, 1.24 mm, 1.16 mm, respectively. The average rotation positioning error in the X, Y, and Z direction of control group and experimental group were 1.29°, 1.02°, 1.01° and 1.08°, 0.96°, 1.00°, respectively. The translational setup errors in the Y and Z directions and rotational setup errors in the X direction significantly differed between the control and experimental groups (all *p*<0.05), but no statistical significance was found in the other directions (all *p*>0. 05).

**Conclusion:** Compared to the traditional thermoplastic head membranes, 3D printing head membranes has shown a reliable and reproducible interactional positioning accuracy. Of course, further investigations are needed before the new technology can be used on a regular basis.

## Introduction

Thermoplastic head film is the most commonly used postural fixation tool in cranial radiotherapy 1. However, the material typically used to produce thermoplastic head film tend to shrink when cooled, resulting in isocenter deviations and thus reducing the accuracy of treatment. Further, thermoplastic head film used to position the patient is often too flexible and hence insufficient to immobilize the patient's head 2. In addition, as individual patients vary greatly, the unified thermoplastic head film often fails to match the patient's individual anatomical head characteristics, further reducing the precision of head fixation. Though the most commonly used thermoplastic head film is a reliable and safe immobilization device, it is prone to trigger stress or fear. According to Nixon et al., approximately 26% of patients suffer from anxiety concerning the thermoplastic head film 3,4. In our experience, even non-claustrophobic patients experience problems as they often report that the thermoplastic head film is uncomfortable. Especially pressure or even pain in the area of the nose or forehead is often described. Altogether, several factors render the use of thermoplastic head film suboptimal for patients who undergo multiple sessions and require precise head position fixation.

The emerging 3D printing technology known as “additive manufacturing” creates 3D structures through successive layers of material from the bottom up 5,6, instead of cutting or milling out the shape of the object from a larger volume of material, or casting molten material in a mold. Because of its low-cost and ease of use, this technology has widespread application in various industries. 3D printing technology has tremendous potential for development in medical care, with the numerous new research demonstrating the ability of 3D printing technology to drive innovation 7-9.

3D printing technology is also of great clinical value in the field of radiation oncology, both for quality-assured phantoms and brachytherapy applications, as well as for beam modulators and boluses 10-13. Individualized 3D printing technology offers many advantages over the traditional thermoplastic: Above all, the more uniform thickness reduces ray scattering and avoids hot and cold spots 14, 15. Next, the area covered by the 3D printing technology is more accurate; reducing unnecessary dose increases to distant organs.

Preclinical research focused on the selection of materials and fabrication of 3D printing technology, as well as preliminary dosimetry validation on the human body model. 3D printing technology has little practical experience in the field of radiotherapy with small sample size and limited disease types 16. In this study, 3D printing technology was used to personalize precise radiotherapy head films for cranial tumor patients. In order to ensure the accuracy of research, aim and value, we had enrolled more patients, expanded disease types and applied new materials. We compared the effect of the material on deep dose attenuation and body surface dose between this new approach and the use of conventional thermoplastic head film. Further, positioning accuracy and repeatability were evaluated in clinical application.

## Materials and Methods

### Clinical case selection

This study was approved by the institutional review board at our institution (No. 2019016). The need for informed consent from each patient was waived by the institutional review board because this study was non-invasive and utilized routine treatment data based on patient data confidentiality and compliance with the Declaration of Helsinki. From September 2017 to September 2019, thirty patients with head and neck radiotherapy (13 patients after glioma operation, 6 patients with hypopharyngeal cancer, 6 patients with parotid cancer and 5 patients with meningioma operation) were selected from our radiotherapy center, including 17 male patients and 13 female patients, the median age was 59 years (range 45 to 73), and KPS ≥ 70. The characteristics of all patients are shown in Table 1. Fifteen cases in each group were divided into the control group and the experimental group. The control group used conventional thermoplastic head film for head position fixation, while the experimental group used 3D printing technology to print personalized head film for patients for body position fixation. An intensity-modulated radiotherapy plan was developed for both groups, with prescribed dose of 45.0 - 69.96Gy, divided into 25-33f, 5f a week.

### Membrane material and structure

The thermoplastic head membrane plate was made of synthetic high molecular polyester with a density of 1.12 g/cm^3^ and a mesh-like structure. The thickness of the human head membrane after stretching ranged from 1.5 mm to 1.9 mm, with an average of about 1.7 mm. Template dimension for dose testing was 200 mm ∙ 200 mm ∙ 1.7 mm. The 3D printing head membrane used for clinical tests was made of epoxy resin, with a density of 1.076 g/cm^3^ synthetic resin, 3D printing size and thermoplastic head film consistent (200 mm ∙ 200 mm ∙ 1.7 mm) template for dose test. The 3D printing head film for clinical application is made of epoxy resin with uniform holes.

### Epoxy resins

Epoxy resins are classified under the name of ethoxyline resins. These resins, which are produced by the condensation of bisphenol A and epichlorohydrin, contain terminal epoxy groups and may contain many hydroxyl pendant groups, depending on molecular weight 17.

### Production of cephalic membrane

**Figure 1:**
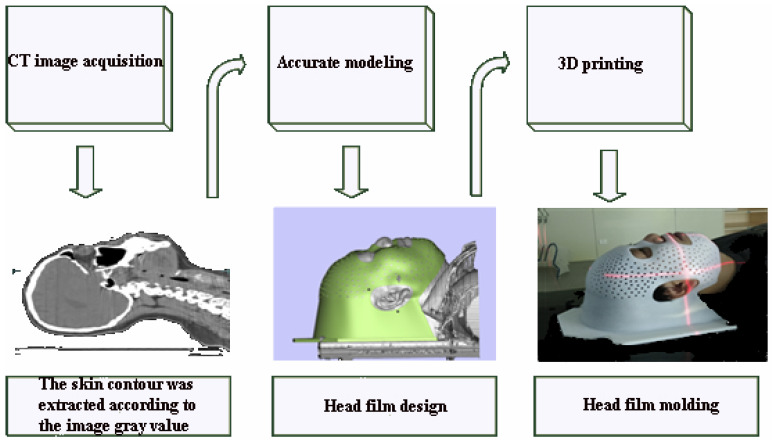
3D print head film production process.

The patients in both groups were in supine position. Positioning CT scans were performed using a standardized headrest combined with a head rest in a fixed position according to the patient's actual condition: a. In the control group, softened "U" thermoplastic film was heated (approximately 60-70oc) and covered over the patient's head, cooled and shaped by manual stretching and pressure to create the cephalic membrane, b. In the experimental group, CT scans of the patient's head (reconstruction layer thickness 1.5 mm) images were transmitted to the 3D printing image processing platform in the standard digital imaging and communications in medicine (DICOM) format for 3D modeling. After setting thresholds, the skin contours were extracted according to the image gray value and the printing range was marked by the spline curve. The stereolithography (STL) file isoplanes were sliced in the material magics, and the 3D printer used the light curing technology for entity printing. The design and printing time of 3D printing individual head film was generally 4-6 hours (Figure 1).

### Beam device and measuring equipment

A Synergy linear accelerator produced by Elekta was used for the present study. The beam energy was a conventional 6MV x-ray (with uniform mode) with a dose rate of 600 MU/min, the beam size was 10 cm ∙ 10 cm, and the accelerator beam flow was 200 MU. An FC-65G finger ionization chamber and solid water model produced by the IBA were selected for the dose attenuation test in depth. The same batch of films and solid water after the dose calibration were selected for the body surface dose test 18.

### Deep dose attenuation test

The ionization chamber was inserted into the solid water model with the measurement depth set at 5 cm and the source skin distance (SSD) set at 95 cm. The dose under the cover of the thermoplastic head film template and 3D printing head film template on the surface of solid water mold was measured and compared without the cover to evaluate the degree of attenuation.

### Body surface dose test

The film was placed flat on the surface of solid water, the SSD was 100 cm. Next, we measured the dose under the film covering the thermoplastic head film template and the 3D printing head film template, respectively. The obtained values were compared without the covering to assess the effect of the water mold surface position and the material on the dose.

### Clinical case evaluation

The 30 patients underwent kV-cone beam computed tomography (kV-CBCT) verification imaging during their first treatment every week. Each patient was initially positioned based on marked points on the patient's head. The patients then underwent a 360-degree verification kV-CBCT. The kV-CBCT image study set was automatically registered to the planning image study set and manually fine-tuned using bony marks and the outer contour of the skin to obtain the translation data in the X (left and right), Y (superior and inferior) and Z (Anterior and posterior) directions as well as the rotation data in the 3 axes.

### Statistical method

SPSS 22.0 software was used to analyze the positioning error of CBCT. The measurement data is reported as 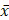 ± s (mean ± standard deviation). Using the independent sample t tests and a level of P < 0.05 was considered statistically significant.

## Results

### Deep dose attenuation measurement results

At the depth of 5 cm, the attenuation rate of the two materials was less than 1% (P > 0.05), indicating that both materials have good X-ray transmission.

### Body surface dose measurement results

In the surface position, the surface dose increased by approximately 27% after stretching using a thermoplastic head film with the thickness of 1.7 mm. Using a 3D printing head film with a print thickness of 1.7 mm, the body surface dose increased by about 18%. The lower surface dose of the 3D printing head film compared to the thermoplastic head film suggests that 3D printing material provides better protection on the skin as it produces relatively fewer scattering lines at the tissue interface (Table 2).

### Patients positioning error measurement results

**Figure 2:**
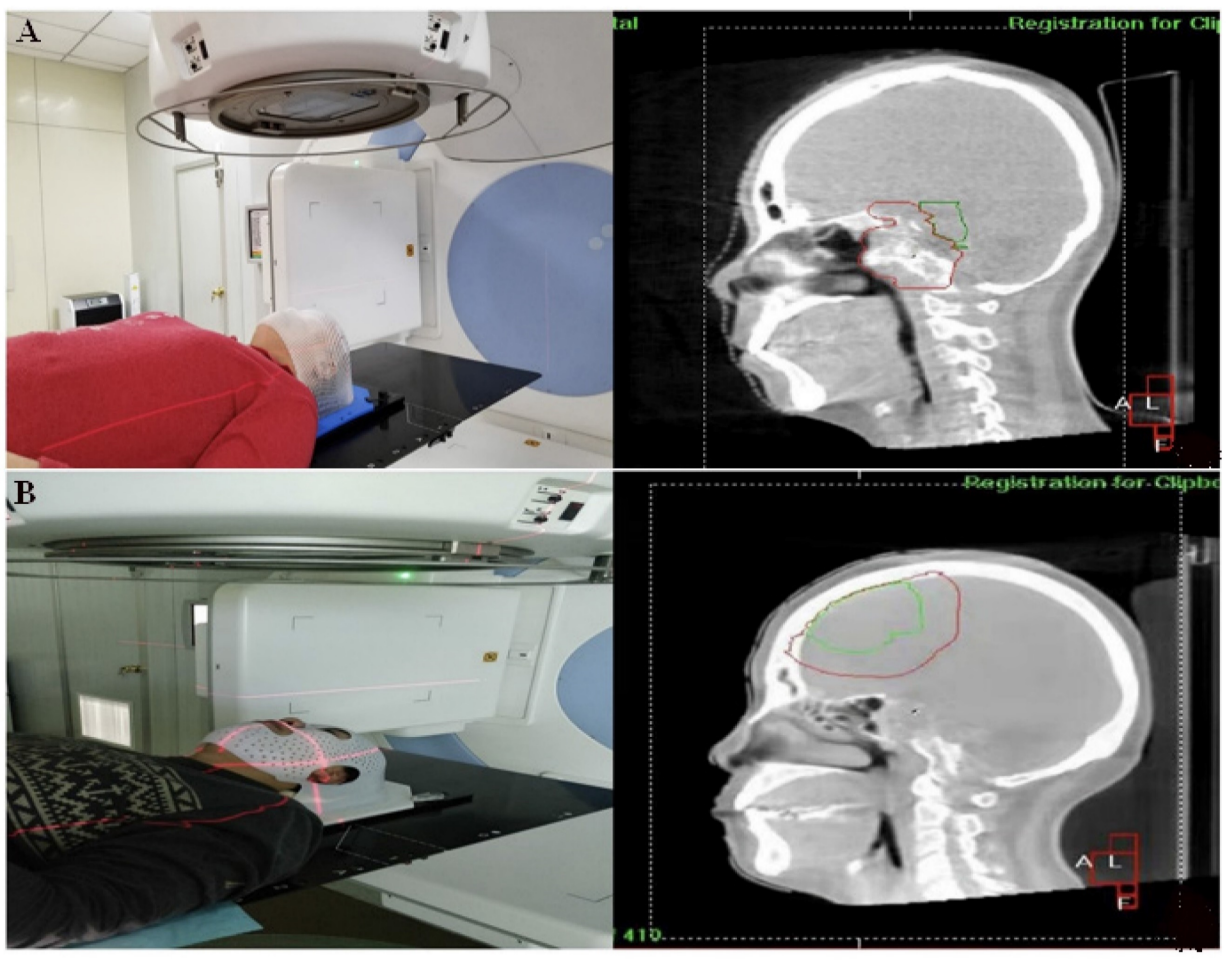
Patients treated with the thermoplastic head membrane plate and kV-CBCT images (A) and patients treated with 3D printing head membrane and kV-CBCT images (B).

**Figure 3:**
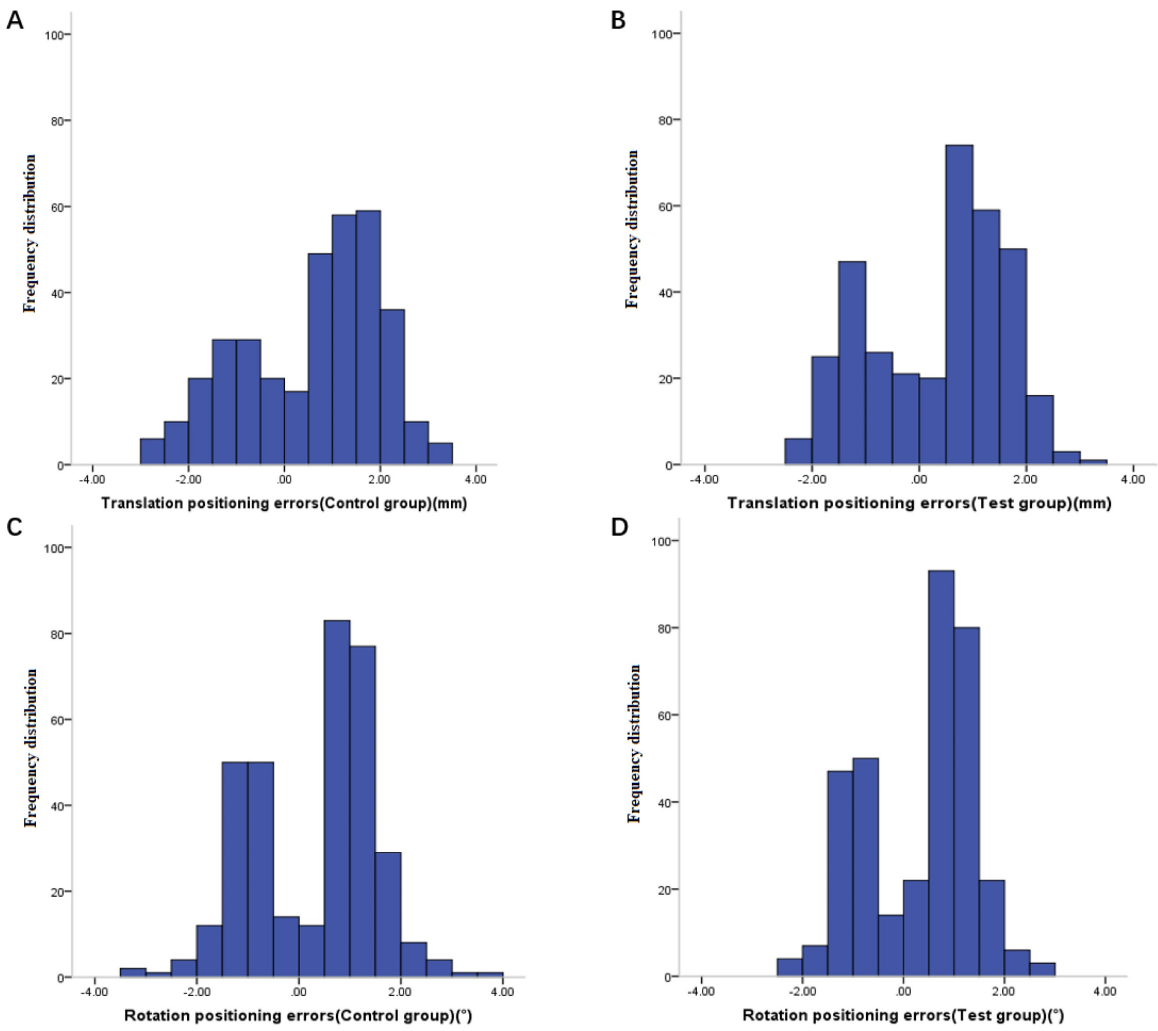
Frequency distribution of translation positioning errors in control group (A), frequency distribution of translation positioning errors in experimental group (B), frequency distribution of rotation positioning errors in control group (C) and frequency distribution of rotation positioning errors in experimental group (D).

After kV-CBCT posture verification (Figure 2), 116 groups of positioning errors data were taken from each group (Figure 3). The mean translation and rotation positioning errors of control group in three directions were 1.36 ± 0.67 mm and 1.11° ± 0.48°, respectively. The average value of translation and rotation positioning errors of experimental group in three directions was 1.18 ± 0.55 mm and 1.01° ± 0.45°, respectively. Compared with the control group, the translation and rotation positioning errors in different directions in the experimental group were reduced. The translation errors in Y and Z directions and the rotation errors in X direction were statistically significant (P < 0.05). Moreover, the translation error in X direction and the rotation errors in Y and Z directions were similar to those in the thermoplastic head film with a trend of decreasing effect, however, tests results did not reach statistical difference (P > 0.05) (Table 3).

## Discussion

Accurate and reliable postural fixation technology is an important development in achieving accurate postural fixation and are therefore key to the delivery of high-quality radiotherapy in patients. However, the human head is a relatively complex anatomical structure, which makes it difficult to apply therapy dosages in a spatially precise manner. Moreover, tumors are often in close proximity to vital organs and brain areas, which need to be protected. For instance, ICRU Report No. 24 pointed out that 3% - 5% of the dose change will cause the primary focus out of control or result in treatment complications 19. Hence, little variation is therefore important and it remains pivotal to provide precise head positioning and that is comfortable for patient and reliable for multiple courses of treatment. During the manufacturing process of the traditional thermoplastic head film, various factors can lead to shrinkage and deformation of the material used, including physical stresses on different surfaces, insufficient cooling time at production stage and different storage conditions in the later stage. These factors predispose patients at head displacement from the location mark line, which negatively affects the accuracy and therefore the effectiveness and safety of the radiotherapy treatment. At the same time, the “nodding” movement of the cervical spine is a unique non rigid movement of the head and neck. Although conventional thermoplastic head film for body position fixation used in radiotherapy can partly compensate for this effect, the materials used are relatively soft and therefore do not adequately absorb this movement. Moreover, hyperextension of the mandible can lead to poor fixation effect and large rotation error. For example, Polat *et al.* 2 found that the maximum rotation error of left-right axis can reach 11° when the thermoplastic head film is used to fix the body position. For radiotherapy of head tumors, a small rotation error already has a substantial impact on the dose distribution that arrives at the tumor target 20. In addition, the thermoplastic head film needs to be stretched downwards with the thermoplastic film to hold the patient in the simulated positioning bed and in the same position for a long period of time during the forming of the head film. During this process, the patient's mouth and nose are subjected to pressure, which results in poor comfort and high compliance requirements. Hence, a considerable number of patients likely have to contend with additional, preventable mental pressure prior to radiotherapy, which further increases the patient's physical discomfort 21.

Sanghera *et al*. 22 first proposed the concept of customizing cephalic membranes for head radiotherapy patients using a digital platform with an optical scanner. Later, Mackernan *et al*. 23 used a laser surface scanner combined with numerical control technology to customize the cephalic membrane for patients suffering from head tumors using a milling machine, however, this approach was not further promoted due to high production costs. With the introduction of 3D printing technology into the medical field, Laycock *et al.* 24 demonstrated the feasibility of applying 3D printing technology to the production of radiotherapy head film, testing the physical dose characteristics of different 3D printing materials. Robertson *et al*. 25 and others used 3D printing technology to make individualized head film for 17 volunteers and evaluated the repeatability of head film placement through the displacement of marking lines on the head film in different directions. They further evaluated the experience reported by patients. Their results showed that the positioning accuracy of the 3D printing head film met the standards for use in clinicals and that the production and use of the head film did not result in adverse psychological effects in patients. However, to date there are very few reports on the use of this method in the clinical medicine, especially in radiotherapy. Mattke *et al*. 16 explored the feasibility of 3D-printed fixation masks for whole brain radiation therapy in a clinical setting and performed a first comparison to an established thermoplastic mask system. Six patients were irradiated with whole brain radiotherapy using individually 3D-printed masks. This study showed a reliable and reproducible interfractional positioning accuracy using individually 3D-printed masks for whole brain irradiation in a clinical routine. A systematic review was conducted across thirty-eight databases by Asfia *et al*. 26. A total of eighteen papers suitably detailed the use of 3D printing to manufacture and test immobilisers, and were included in this review. It was found that a lack of technical knowledge, combined with disparate studies with small patient samples, required further research in order to validate claims supporting the benefits of 3D printing to improve patient comfort or treatment accuracy. However, small sample size trials might have biased results, although no significant effect of publication bias was detected. Hence, we used 3D printing technology to customize personalized radiotherapy head film for cranial radiotherapy patients. We expanded the sample size and disease types of this study to demonstrate the dosimetric characteristics of its material and the treatment positioning accuracy.

The results of this study show that the rotation positioning errors in X direction of patients using 3D printing individualized head film are significantly improved. Further, the translation and rotation and positioning errors in other directions were not significantly improved, but there was a trend for an effect of reduction. The positioning accuracy of 3D printing head film meets the positioning requirements of modern precision radiotherapy and is consistent with the traditional body position fixation technology. The 3D printing head film uses epoxy resin as the printing material. The cured material is hard, non-toxic, milky white, odorless after post-treatment, with a smooth surface, good skin affinity, and low shrinkage at room temperature. In the present study, the head films with thickness of 2.0 mm, 1.7 mm and 1.0 mm were printed respectively (when the thickness is less than 1 mm, the printing effect is poor). In order to be consistent with the traditional thermoplastic head membranes, the head films with a thickness of 1.7 mm were selected as the printing thickness, and holes were punched evenly during the production of the head films, so as to reduce the impact of materials on the completion effect. The dose on the surface of the body after covering the thermoplastic head film was 2.4 times higher than that without the film, a finding that is consistent with that reported by Hadley *et al*. 27. The attenuation of energy and the dose on the surface of the 3D printing material after drilling were equivalent to that of the thermoplastic head film. In the process of making 3D printing head film, patients only needed to carry out plain CT head scan, which circumvents the potential psychological and physical discomfort that patients are prone to during conventional thermoplastic head films. Moreover, modeling and printing are highly automated and therefore a potentially affordable process.

Because of the 3D printing individualized head film design and further possibilities in individualization (e.g., bigger holes for eyes, mouth, and nose), the new 3D printing individualized head film has potential to improve patient comfort compared to the traditional thermoplastic head membranes 3, 4. In addition to that, the uncomfortable part of pulling the wet blank onto the patient's face is redundant during the production process. Especially patients who suffer from claustrophobia could benefit from those features. As the current study was a pilot project focusing on practicability, no standardized assessment concerning patient comfort has been carried out. This will be subject to further investigations.

Additionally, the creation process can be automatized, as the patients don't need to be present in the creation process, as is the case with the traditional thermoplastic head membranes. Once the required information has been obtained, the head film can be printed and will be ready when the patients arrive for their planning CT scan. This could be clinically beneficial as the time intervals for the planning CT scan can be shorter than that with the creation process of the traditional thermoplastic systems.

As it was a pilot project, the overall clinical workflow was more complex than the established one using traditional thermoplastic. We are well aware of those limitations and are currently experimenting with new methods to optimize the workflow by using optical scanners. Like that, the patient's information could be acquired in the outpatient department already, the head film could be produced automatically and, afterward, the CT scan could be acquired all in one appointment. There are some weaknesses in our study. First, a detailed cost-effectiveness analysis has not been performed as part of our study, which we will investigate in the future. Second, we need to evaluate more different target volumes and disease types. Therefore, it is necessary to evaluate more patients. Last but not least, 3D printing offers a wide range of possibilities considering individual positioning of otherwise that are difficult to fixate body parts like arms or legs. This will be subject to further research.

## Conclusion

In short, the current study uses a 3D printing head film in an everyday routine patient treatment. The new technology has shown a reliable and reproducible interactional positioning accuracy. Of course, further investigations are needed before the new technology can be used on a regular basis.

## Figures and Tables

**Table 1:** Patient Characteristics

Characteristics	3D print head film n=15, n (%)	Traditional thermoplastic head film n=15, n (%)
Media age	58	60
Gender		
Male	9 (60)	8 (53)
Female	6 (40)	7 (47)
Headrest type		
Type B	13 (87)	14 (93)
Type C	2 (13)	1 (7)
Treatment fraction (mean)	28.6	29.3
Treatment dose (Gy) (mean)	58.3	57.6
Diagnosis		
Glioma	6 (40)	7 (47)
Hypopharyngeal cancer*	3 (20)	3 (20)
Parotid cancer	3 (20)	3 (20)
Meningioma	3 (20)	2 (13)

* Use of head and neck masks in patients with hypopharyngeal cancer.

**Table 2:** Dosimeter value and film value

Test conditions	The dose at the depth of 5 cm (cGy)	The attenuation rate (%)	The surface dose (cGy)	Percent depth dose in surface position (%)
No coverage	186.70	0.00	37.07	18.53
Using a 3D printhead film	184.97	0.93	73.55	36.78
Using a thermoplastic head film	185.63	0.57	90.62	45.30

**Table 3:** Translation and rotation positioning errors in two groups (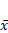 ± s)

Groups	No. of patients	Translation positioning errors (mm)			Rotation positioning errors (^◦^)		
		X	Y	Z	X	Y	Z
Control group	15	1.29±0.61	1.42±0.75	1.38±0.64	1.29±0.59	1.02±0.37	1.01±0.47
Experimental group	15	1.16±0.59	1.24±0.55	1.16±0.52	1.08±0.46	0.96±0.43	1.00±0.45
P value		0.21	0.04	0.02	0.04	0.16	0.34

## Abbreviations

SSD: Source skin distance
ICRU: International Commission on Radiation Units & Measurements
CBCT: Cone beam computed tomography
STL: Stereolithography
DICOM: Digital Imaging and Communications in Medicine
